# Effects of the physicochemical properties of gold nanostructures on cellular internalization

**DOI:** 10.1093/rb/rbv024

**Published:** 2015-12-03

**Authors:** Ningqiang Gong, Shizhu Chen, Shubin Jin, Jinchao Zhang, Paul C. Wang, Xing-Jie Liang

**Affiliations:** ^1^CAS Key Laboratory for Biological Effects of Nanomaterials and Nanosafety, National Center for Nanoscience and Technology, Beijing 100190, China;; ^2^Key Laboratory of Chemical Biology of Hebei Province, Key Laboratory of Medicinal Chemistry and Molecular Diagnosis of the Ministry of Education, College of Chemistry and Environmental Science, Hebei University, Baoding 071002, P. R. China and; ^3^Laboratory of Molecular Imaging, Department of Radiology, Howard University, Washington, DC 20060, USA

**Keywords:** gold nanostructures, cellular internalization, size, shape, surface chemistry

## Abstract

Unique physicochemical properties of Au nanomaterials make them potential star materials in biomedical applications. However, we still know a little about the basic problem of what really matters in fabrication of Au nanomaterials which can get into biological systems, especially cells, with high efficiency. An understanding of how the physicochemical properties of Au nanomaterials affect their cell internalization is of significant interest. Studies devoted to clarify the functions of various properties of Au nanostructures such as size, shape and kinds of surface characteristics in cell internalization are under way. These fundamental investigations will give us a foundation for constructing Au nanomaterial-based biomedical devices in the future. In this review, we present the current advances and rationales in study of the relationship between the physicochemical properties of Au nanomaterials and cell uptake. We also provide a perspective on the Au nanomaterial-cell interaction research.

## Introduction

Materials in nanoscale usually exhibit some unique properties in physical and chemical aspects compared with bulk materials. Rapid development of nanoscience and technology promises fundamental changes to a wide range of research fields such as energy conversion and storage, catalyze, sensing, drug delivery and imaging. Among these applications, Biomedical applications of nanomaterials have attracted the most attention in the past two decades and a number of nanomaterials have been developed for disease diagnosis and therapy [1–3]. Generally speaking, there are two kinds of nanomaterials that have been evolved for biomedical applications. One is organic materials such as liposome, micelle, vesicle, etc. Another group is inorganic materials including carbon nanotube, graphene, gold nanomaterials, quantum dots and silica-based nanomaterials. Each material has its unique characteristics and can be utilized for different purposes [4–6]. Organic materials possess better biocompatibility and biodegradability [7, 8] compared with inorganic materials. However, inorganic nanomaterials are often better in stability and synthetic controllability [9] Among various of materials, gold nanostructures showed great potential in biomedical application as their non-toxic and nonimmunogenic nature, facile fabrication, controllable size and shape, as well as ease of surface modification [10,11]. In addition, the unique optical properties of gold nanostructures, which were known as surface plasmon resonance (SPR), are highly shape- and size- dependent, All of these unique features make Au nanomaterials promising candidate for biomedical applications such as drug delivery, photo-thermal therapy, photoacoustic imaging and so on [12–15].

As we all know, basically all of the biological events are mostly based on material and cell interactions. As different gold nanomaterials can be constructed with variations in sizes, shapes and surface chemical properties, the interactions between cells and nanomaterials differ in thousands of ways. Researchers from nanotechnology and biology field have done tremendous studies on the cellular internalization of gold-based nanomaterials [15–18]. However, a conclusion is still missing as a result of too much contrary research works. In this review, we will provide an overview on how the size, surface chemistry and shape of nanostructure will affect the uptake of gold nanostructures into cells. The aim is to provide some useful information for further studies.

## The effect of size on cellular uptake

Nanomaterial means the size of material is ranged from 1 to 1000 nm. As mentioned as earlier, material in nanosize exhibits many different properties in physical and chemical aspects compared with bulk materials. Moreover, size also matters within nanoscale because many works have proved the size-dependent manner in the behavior of nanoparticles. A small change in size will lead to large deviation results. One key point in nanotechnology is to clarify the size effect of nanoparticles. Gold nanoparticle (Au NP) is one of the promising nanomaterials for the biomedical application. The size effect was also pointed out by countless works. Researchers from nanobiology field share a consensus that size plays a dominant role in the interaction between Au NPs and bio interfaces (including vessels, cells, organelles, etc.). But a conclusion continues to be missing as a result of too much contrary research works. There have lots of factors will affect the materials’ cellular internalization. Here, we are going to discuss how the size determines the internalization, biodistribution of Au NPs *in vitro*.

Internalization of Au NPs by cells is the fundamental process when utilizing Au NPs as drug carriers or contrast agents. Au NPs are internalized by cells in a size-dependent manner. Trono *et al.* [19] reported 20 nm is the best size of Au NPs for cellular uptake. 10, 20, 30, 40, 50 and 100 nm Au NPs were successfully synthesized and characterized. Three pancreas cancer cell lines were incubated with as-synthesized Au NPs. Au amount per cell was then measured and calculated after incubation. 20 nm Au NPs treated pancreas cancer cells show the highest Au amount per cell compared with that of other Au NPs treated cells. However, 20 nm may not be the optimal size according to Chan *et al.* They studied the size dependence of Au NP uptake into mammalian cells and suggested that 50 nm Au NPs were internalized faster in speed and greater in amount than 14, 30, 74 and 100 nm Au NPs [17]. The uptake half-life of 50 nm Au NPs is 1.90 h, shorter than that of 14 nm (2.10 h) and 74 nm (2.24 h).

These studies make other researchers confused and leave them at a loose end. Which one can be trusted and applied as standard? We need a conclusive result to guide the design of Au NPs when utilizing them. However, we should be aware of that it is impossible for us to come to a rule covering all the conditions. Another work from Chan’s group may help us to understand this situation [20]. The purpose is to study the cellular uptake after particle aggregation. Aggregated Au NPs were produced by disrupting electrostatic forces between particles. Cells were therefore incubated with aggregated Au NPs and cellular uptake was identified. Both Hela and A549 cells exhibited a 25% decrease in uptake of aggregates compared to that of monodispersed Au NPs. However, another cell line, MDA-MD-231, showed a 2-fold increase under the same treatment. This means a case-by-case basis should be followed while we are studying the behavior of atypical Au NPs. Based on the above work, we should also notice that aggregation is a factor which will change the interaction between cell and nanoparticles. Aggregation occurs once repulsion force (like electrostatic repulsion) between particles is compromised in a complex situation with ions, proteins or undesired pH value for stability of particles [21]. When nanoparticles are applied in a physiological environment like blood or culture medium, most of the contents in the environment will lead to aggregation and should be considered when interpreting the results. Net charge and polymer modification are often employed to prevent aggregation but no insurance can be made whether they can stop aggregation or not. Size increase was always observed after nanoparticles were suspended in solutions. Some of the increase should be attributed to hydrodynamic diameter which means particle would be larger in solution than in dry state. However, some unreasonable size change in solutions should be discriminated whether it is caused by aggregation. In the case of size effect, whether the size-dependent manner is a result of size or it’s caused by different levels of aggregation should be concluded by monitoring the size change and stability of particles before and after they are introduced to the physiological environment.

**Figure 1:**
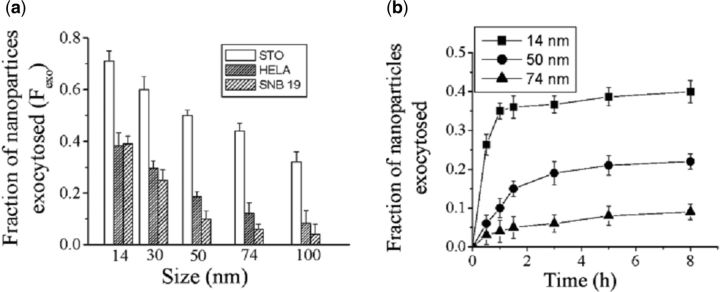
Size-dependent exocytosis of Au NPs. **(A)** Exocytosis fraction of 14–100 nm Au NPs in three cell lines. **(B)** Kinetics of Au NP exocytosis. Adapted with permission from [16], Copyright 2007 American Chemical Society.

Exocytosis is another key process related to the accumulation of Au NPs in cells. Exocytosis rates of different Au NPs were measured by Chan and co-workers [16] in their study. They found that Au NPs were exocytosed in a linear manner in size. Smaller Au NPs exhibited quicker exocytosis rate and percent. 40% of 14 nm Au NPs were finally exocytosed while >90 % of 74 nm Au NPs were remained in cells after 8 h (Fig. 1).

Distribution of Au NPs in cells is highly relevant to the biological effect and further application of Au NPs. Our group reported the relationship between the accumulation of Au NPs in lysosomes and autophagy process [22]. Au NPs were found to be trapped in lysosomes after internalization and lysosomes were proved to be swelled and alkalized with Au NPs inside. The alkalized lysosomes could not fuse with autophagosomes which will lead to the blockade of autophagy. Thus it is important to figure out the distribution of Au NPs in cells before utilizing. Size also matters relating to the intracellular distribution of Au NPs. A study about the cellular uptake and fate of PEGylated Au NPs functionalized with cell penetrating peptide (CPP) suggested that size determined the distribution [23]. 2.4 nm Au NPs were found in nucleus while 5.5 and 8.2 nm Au NPs could not achieve intra-nucleus distribution and were partially located in the perinuclear site. Other 5.5 and 8.2 nm Au NPs were located at the cellular peripheral. Another study work for ultrasmall Au NPs also demonstrated the superior penetration of smaller Au NPs in cells and in tissues [24]. 2 and 6 nm Au NPs were distributed throughout the cytoplasm and nucleus in cells and penetrated deeply in tumor tissue model. Meanwhile, 15 nm Au NPs were only found to be in cytoplasm and could not achieve deep penetration compared with 2 and 6 nm Au NPs. Intra-nucleus distribution of ultrasmall Au NPs enables them to be applied as nanocarriers of therapeutic agents to nucleus. Wei and co-workers also reported an interesting phenomenon in the distribution of Au NPs by using plasmonic scattering images [25]. 45 nm Au NPs were internalized through endocytosis as a result of which Au NPs were distributed in endocytic vehicles. Larger Au NPs with 75-nm diameter were imaged to be mainly distributed on the top of cells. These consequences enlighten us to choose size of Au NPs according to the purpose of application so optimal outcome could be achieved.

**Figure 2:**
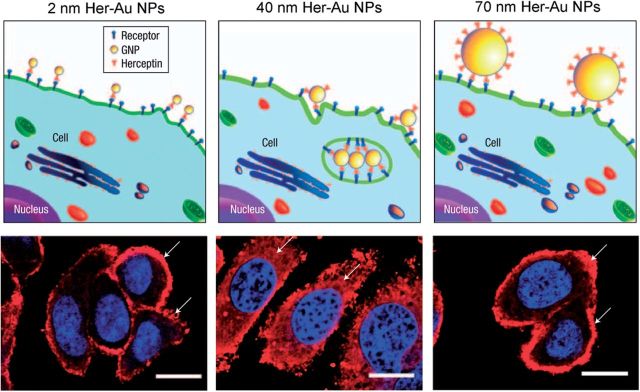
Size-dependent receptor-mediated internalization of Au NPs. Reproduced with permission [27] Copyright 2008, Nature Publishing Group.

Until now, we have observed many size-dependent behaviors of Au NPs as shown earlier. Numerous researchers have therefore contributed their effort to reveal the mechanisms of the size-dependent manner. McNeil *et al.* [26] inspired us to interpret from size-dependent protein binding. Au NPs with diameter of 50 and 30 nm were incubated with human blood. Au NPs were then collected and surface-bound proteins were harvested and characterized by mass spectrometer. The results show that 21 kinds of proteins were bound to 50 nm Au NPs while 48 kinds of proteins, more than 2-fold of that in 50 nm Au NPs, were bound to 30 nm Au NPs. They share 15 proteins which were found to be bound to both 30 and 50 nm Au NPs. According to this research, proteins bound to Au NPs may lead to the different behaviors of Au NPs. Chan *et al.* demonstrated size itself also have a great effect on internalization of Au NPs [27]. They functionalized different sized Au NPs with Herceptin, a ligand of cell membrane receptor ErbB2. Au NPs in this study then shared the identical surface modification. SK-BR-3, an ErbB2^+^ cell line, was treated with as-prepared Herceptin-Au NPs. The internalization of Au NPs was observed by imaging the location of ErbB2 as ErbB2-mediated endocytosis will lead to its distribution in cytoplasm. The result suggested interaction between Herceptin-Au NPs and receptor ErbB2 was strongly size-dependent because 40 nm Herceptin-Au NPs incubated cells exhibited much more ErbB2 distribution in cytoplasm. Size determines the curvature of Au NPs, which is important for surface potential and particle-membrane/receptor interaction. Smaller size means higher curvature and surface potential, as a result of which particle will be less stable. Meanwhile, smaller size will lead to smaller interact area and limited receptor bound on cell membrane (Fig. 2).

## The effect of shape on cellular uptake

**Figure 3:**
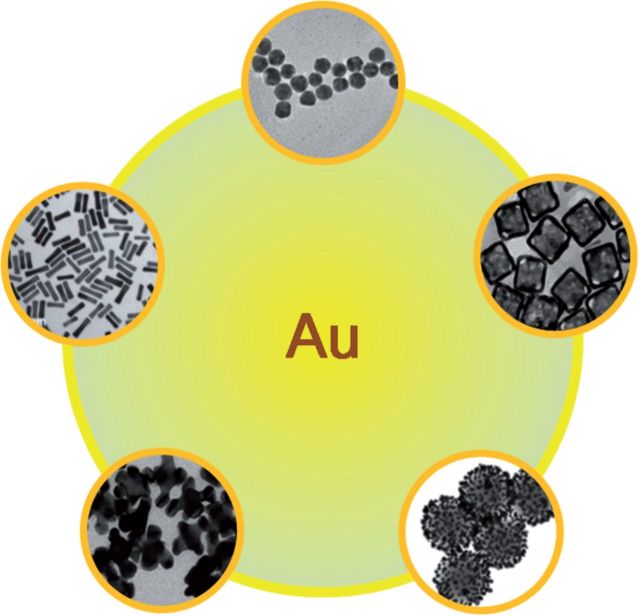
Au based nanomaterials with different structures. The picture of Au nanocage and nanohexapods Reproduced with permission of [58] Copyright 2013, American Chemical Society; The picture of nanoparticles Reproduced with permission [59] Copyright 2011, American Chemical Society; The picture of nanorods Reproduced with permission [60] Copyright 2014, The Royal Society of Chemistry; The picture of nanoshells Reproduced with permission [61] Copyright 2005,American Chemical Society.

As is known to us, gold based nanoparticles can be synthesized with diverse shapes, such as nanosphere, nanorod, nanocage, nanoshell or other irregular structures (Fig. 3). Each kind of material has its unique physiochemical properties and can be implemented in different areas of biological field. For instance, Au nanosphere can be synthesized with ultra-small size and can be used as drug or gene delivery carriers [28, 29]. Gold nanorod with the localized SPR character can be applied in photothermal therapy [30–32]. Gold nanoshells with near-infrared absorption property have been used as photoacoustic imaging contrast agents [33, 34]. However, all these applications are based on the nanomaterials internalization into cells, efficient cell uptake is a prerequisite for gold nanostructures to function as a therapy or diagnostic agents. As mentioned earlier, the size of the Au nanomaterials will affect cellular internalization of these materials. How the different geometries of these nanostructures will impact their uptake by cells?

In addition to the size-dependent effect of cellular internalization, shape also plays an important role in the cellular uptake. Wang *et al.* [35] used different gold nanostructures with the same surface modification (PEGylated) to study the difference of uptake by cells between different nanostructures. They compared the cellular uptake of nanorods, nanohexapods and nanocages and found that after incubations, the cellular uptake of PEGylated nanohexapods by breast cancer cells (MDA-MB-435) was higher than nanorods and nanocages. Their results indicate that the branched morphology of nanostructures might have a higher possibility to enter the cell in comparison with the rod- and/or cube-like nanostructures.

Chan and his co-workers have incubated Hela cells with gold nanostructures with various shapes and calculated the number of nanostructures by the measurement of ICP-AES. The results indicated that gold nanostructures uptake is dependent on the shape and the uptake of rod-like Au NP is lower than the spherical-nanoparticles [17]. The causes of this phenomenon may be caused by the difference in the curvature of rod-like and spherical-like nanoparticles. For instance, the rod-like nanoparticles tend to have a higher contact area with cell membrane receptors than sphere-like nanostructures when the longitudinal axis of the rod contact with the cell membrane. This will lead the reduction of the number of available receptor sites for binding. Another reason was that surfactant (Hexadecyl trimethyl ammonium Bromide, CTAB) will be absorbed onto the rod-shaped nanoparticles during synthesis. The absorption surfactant will block the binding site of serum protein or receptor [36]. The second explanation has been proved by Huff *et al.* [37]. They utilized *in situ* dithiocarbamate formation replace the CTAB surfactant with PEG chains and found the intracellular uptake is also at a lower level. Besides that, Chan and his co-workers also found that within the same surface chemistry, the cellular uptake of rod-like structures with a lower aspect ratio (1:3) is higher than higher aspect ratio (1:5) nanoparticles [17]. In another study the same results were achieved. Arnida *et al.* use macrophages as the cell model to explore the difference between rod- and spherical- like structures on cellular internalization [38]. The results also show that gold nanorods were taken up to a lesser extent by cells compared with spherical nanoparticles. So the conclusion can be drawn that spherical-particles have a higher possibility of being uptake compare with rod nanostructures.

**Figure 4:**
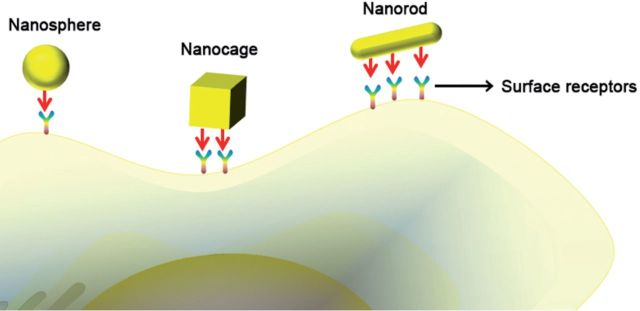
Cross sections of three different shaped gold nanostructure–PEG–antibody (nanosphere, nanocage and nanorod).

Is this conclusion suitable for all situations? The result of course is not. When the nanostructures were modified with targeted ligand, there was a large difference. In an experiment, three nanostructures with different shapes (nanosphere, nanorod and nanocage) were conjugated with antibody for cell targeting. The nanosphere exhibited the lowest cellular interaction with MCF-7 cells. When compared with spheres and cages, rod-like nanoparticles have the highest surface cross-section, which results in higher antibody-mediated cellular uptake (Fig. 4) [39]. The curve shape of spherical particles allows less number of binding sites to interact with target cell receptors and the elongated nanostructures show higher efficiency in adhering to the cells. So the elongated architecture with higher aspect ratio has extensive uptake than the spherical particles with the same size.

Although extensive studies have been done to study the influence of geometry on cellular uptake, but the results are quite different [17, 18, 40, 41]. The general conclusions that can be learning from studies are still preliminary. For instance, with a certain mass, nanostructures with different shape will also have a different volume and numbers. Previous study handled by Parakhonskiy *et al.* [42] indicated that the number of internalized CaCO_3_ nanoparticles depends on the aspect ratio of the particle. When compare with lower aspect ratio particles, particles with a higher aspect ratio show a higher extent of cellular uptake. Interesting, they found that in case cells were exposed to the same amount of particles, the total volume uptaken by cells depends on the volume of individual particles. This conclusion may be also applied to gold-based nanostructures. But there is almost no profound research in this aspect and need more future explore.

There have so many factors such as the type of cells, incubation conditions, the function of size, surface chemistry and the aggregation state will also affect the cells uptake. In the research of uptake of gold-based nanomaterials by cells, we need to consider more comprehensive factors. Besides that in order to successfully apply gold based nanostructures in biomedical application, it is crucial to study the exocytosis and the clearance mechanisms of nanostructures.

## The effect of surface properties on cellular uptake

As the nanoparticle-cell interaction usually is resent at the interface between NPs and cells. The surface properties of Au nanomaterials are considered as the most important factor that affects cell uptake.

Cell membranes are bilayer lipid in structure. Cell membranes usually display a strong negative charge as the phosphate group of lipid. As electrical interactions between negatively/positively charged NPs with negatively charged cell membranes exist, it is obvious that the electrical properties affect cell uptake. Several studies have investigated the effects of surface charge on cell internalization of Au NPs [43, 44], these studies indicate NPs with positive charge are easier to be internalized by cells, but limitations still exist as these studies can’t distinguish the cell membrane adsorbed NPs from internalized NPs. Cho *et al.* [45] developed I_2_/KI etching strategy that can precisely quantify the membrane adsorbed Au NPs and the internalized NPs. They found that the positively charged Au NPs were adsorbed much more on cell membranes and thus have the highest cell internalization amount compared with those of neutral and negatively charged NPs. These studies demonstrated surface charge play a critical role in cell internalization of Au NPs, but does surface charge the sole parameter that affect cell uptake of Au NPs? The answer is no, it is far from simple when Au NPs come into culture media or blood. Recent studies have proved the existence of protein corona when nanoparticles were exposed to culture medium. For instance, Chen *et al.* [46] demonstrated that CTAB-coated Au-nanorod can absorb BSA molecules of culture medium using synchrotron radiation X-ray absorption spectroscopy. They found at least 12 Au−S bonds between Au nanorods and protein molecules formed. Moreover, Alkilany *et al.* [47] revealed that both cationic and anionic gold nanorods can adsorb proteins to their surface within several minutes. The absorbed protein can change the surface charge of NPs immediately to similar negative value as the serum proteins of the medium. Consequently, positively charged NPs are no longer cationic when they are in the culture medium. These works reminded us the protein corona on the surface of Au NPs may be something that directly interacts with cells. Moreover, studies found protein adsorption can influence cell uptake of NPs [48]. For example, Conner *et al.* [49] found protein adsorption can facilitate cell internalization of NPs by receptor-mediated endocytosis. So whether the difference on cell membrane penetrating capacity of cationic NPs and anionic NPs can be explained by the different protein adsorption capacity of these two kinds of NPs? Wolfgang *et al.* [50] demonstrated that there is no difference in the number of the adsorbed serum albumin molecules between the positively and negatively charged Au NPs when these particles are dispersed in culture media. But experiments both in the serum-free and serum-containing media get the same result, that is, the positively charged Au NPs revealed a higher internalization efficiency than the negatively charged NPs. All these studies remind us consider the surface charge properties when constructing nanoparticle-based bio-devices.

In prevention protein adsorption, Rotello *et al.* [51] reported that the surface hydrophobicity can prevent protein adsorption and thus cellular uptake of Au NPs. In their designation, all four kinds of Au NPs contain the same positive charged quaternary amine group, but the different lengths of alkane chain endue NPs varying degrees of hydrophobicity. Cell internalization of these four Au NPs was examined by incubating Au NPs with cells for several hours and analysed by ICP-MS. They have concluded that the presences of serum can significantly reduce the cell internalization of these Au NPs as protein absorption. Moreover, they found that the more the surface hydrophobicity of Au NPs is, the easier protein adsorbs, and in turn the more cellular uptake decrease. Their study tells us the surface hydrophobicity may play a critical role in adjusting serum albumin binding, which ultimately affects the internalization of Au NPs in cells.

Apart from surface charge, surface ligands also play an important role in Au NPs cell internalization. Surface ligand of Au NPs can be peptides, antibodies or oligonucleotides. These targeting molecules can be recognized by cell surface receptors and thus permeating into cells by receptor-mediated cell uptake. CPPs are one type of peptides which usually contain high content of cationic residues. They can physically adsorb to the negatively charged cell membranes or can interaction with cell surface receptors and then CPP-mediated cell uptake can occur through various endocytosis ways. Till now, many kinds of CPPs have been evolved [52, 53] including both cell-type specific and non-specific CPPs. Among various kinds of CPPs, RGD-based peptide has drowned the most attentions as its selective cell membrane penetrating capacity in active-targeted drug delivery. RGD sequences decoration is a popular strategy in promoting of cellular uptake of Au NPs.

**Figure 5:**
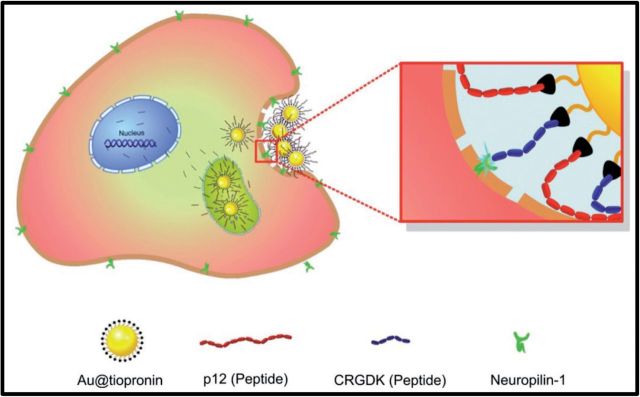
Scheme of designed multifunctional Au NPs with several surface ligands. Reproduced with permission [54]. Copyright 2012, Elsevier.

Our group [54] recently developed CPP-Au based delivery system that can deliver therapeutic P12 peptide with high efficiency (Fig. 5). p12 is a potent inhibitor of MDM2 and MDM2 inhibition results in accumulation of the tumor suppressor p53. p53 is known to transcriptionally regulates a variety of genes in response to many stimuli, which can cause the apoptotic death of cancer cells. In our study, tiopronin modified 2 nm Au NPs were prepared and simultaneously modified with CRGDK peptide and p12 peptide. The CPP peptide can recognize and selectively bind to neuropilin-1 receptors that overexpressed on the tumor cells and ultimately result in receptor mediated cell uptake. The results illustrated that CRGDK functionalization can largely promote cellular uptake of Au@p12 NPs, which can enhance the P12 peptide amount inside the cancer cells. The Au@p12-CRGDK NPs showed great anticancer efficiency by promote p53 level. This design provides a good example for cell type selective drug delivery for cancer treatment.

Aptamers are emerging class of DNA or RNA sequences that serve as targeting ligands or therapeutic molecules. For cellular targeting applications, aptamers can be bind to the surface antigen of cells with high-affinity and selectivity, and entry into cells by receptor-mediated internalization. Recently, Kim *et al.* [55] utilized a prostate-specific membrane antigen (PSMA) specific A10 aptamer to selectively deliver drug-loaded Au NPs to target cells. The aptamer was demonstrated to be capable of selectively binding to PSMA and then enter cells in clathrin-mediated way [56]. The NPs showed great extent cell uptake toward PSMA positive LNCaP cells but not on PC3 cells which is PSMA negative. Moreover, they verified the aptamer-tagged NPs capable of precise imaging and cytotoxicity toward PSMA-positive prostate cancer cells.

Though various targeting ligands have been explored for cell or disease-specific delivery, many problems still exist. Studies demonstrated the targeting molecule may lose their targeting capacity as protein adsorption [57]. Other parameters such as surface rigidity, ligand density affect the cell internalization have been reported. To get the whole picture of the effects of NPs surface properties on cell uptake, there is still a long way to be done. However, we believe if the relationship between various surface properties and cellular uptake are clearly understood, we can ultimately design drug delivery systems with excellent delivery efficiency.

## Conclusion

Au nanomaterials, due to its unique physicochemical properties, have drawn the most attentions for biomedical applications in the past few decades. How can various Au nanomaterials be delivered into biological systems, especially cells, is a basic problem that we must dissolve before these materials come into biomedical market. Though many studies have demonstrated that nanomaterials size, shape and surface properties including surface charge, hydrophobicity, surface ligand, etc. are critical parameters that affect cell uptake, we believe there are still long way to go to get the whole picture of Au nanomaterials-cell interaction. It is an urgent task to do systemic study on the relationship between the physicochemical properties of Au and how these natures affect cell internalization. This study will provide us the criteria for construction of Au nanomaterial-based strategies for biomedical applications both *in vitro* and *in vivo*. Moreover, the advances in Au nanomaterial synthesis, manufacture, surface modification and computer simulation may alter how we fabricating Au-based nanodevices in the future and could lead to novel biomedical applications.
